# The Defense Response of Honeyberry to Root Rot Pathogens: Evidence Based on Pathogen Identification and Host Mechanism

**DOI:** 10.3390/plants14243820

**Published:** 2025-12-15

**Authors:** Siyu Qiao, Dianwen Wei, Hui Chen, Jinghua Yu, Shufang Gong, Zhiyong Niu, Aimin Zhou, Kun Qiao, Jingang Wang

**Affiliations:** 1 College of Horticulture and Landscape Architecture, Northeast Agricultural University, Harbin 150030, China; siyuqiao1997@163.com (S.Q.); 18846087318@163.com (H.C.); shufanggong@neau.edu.cn (S.G.); zyniu001@163.com (Z.N.); aiminzhou@neau.edu.cn (A.Z.); 2 Institute of Natural Resources and Ecology .HAS, Heilongjiang Academy of Sciences, Harbin 150006, China; wdwzrs@163.com; 3 CAS Key Laboratory of Forest Ecology and Silviculture, Institute of Applied Ecology, Chinese Academy of Sciences, Shenyang 110801, China; yujh@iae.ac.cn

**Keywords:** honeyberry, root rot, *Fusarium foetens*, biosynthesis, secondary metabolite

## Abstract

Honeyberry plants (*Lonicera caerulea* L., family Caprifoliaceae), which produce small, highly nutritious berries, have recently been subject to an outbreak of root rot, resulting in a drastic decrease in fruit yield. In this study, we isolated the fungal community from the roots of diseased plants and analyzed the mechanisms of interaction between key fungi and honeyberry plants. Nine fungal morphotypes were identified via observation of cultures and internal transcribed spacer gene sequence analysis. Pathogenicity assays showed that infection with isolates from the genus *Fusarium* reproduced the typical root rot symptoms, and *Fusarium foetens* caused the most severe inhibition of root growth. Transcriptomic analysis of infected plants and Kyoto Encyclopedia of Genes and Genomes pathway analysis revealed that the biosynthesis of secondary metabolites was enriched in roots of honeyberry plants infected with *F. foetens*. This study elucidates that when honeyberry is affected by root rot disease, it produces secondary metabolites to defend against pathogenic invasion, providing a theoretical basis and molecular targets for the green management of this disease.

## 1. Introduction

Honeyberry (*Lonicera caerulea* L.), commonly known as haskap or blue honeysuckle, is a perennial deciduous shrub belonging to the family Caprifoliaceae. It is widely distributed across China, Russia, Japan, and Canada. The leaves and fruits of this species are rich in bioactive compounds. As a medicinal and edible small berry, it has diverse industrial applications and is garnering increasing scientific and commercial interest [1]. The fruit has an exceptional nutritional profile, with anthocyanin content—particularly cyanidin-3-glucoside—several times higher than that of blueberries. The fruit also contains substantial levels of flavonoids, vitamin C, phenolic acids, and essential trace elements [2]. Its antioxidant capacity is significantly greater than that of lingonberry, cranberry, blackberry, strawberry, and others [3,4], underpinning a range of physiological benefits such as anti-inflammatory and lipid-lowering effects [5], neuroprotection and potential antitumor activity [6].

The promising industrial prospects of honeyberry cultivation have driven the development of improved varieties, such as “Beilei” and “Lanjingling” [7], which offer higher yields and better fruit quality without compromising bioactive compound content. “Beilei”, the first approved honeyberry cultivar in China, produces larger fruits with improved taste and higher yield compared with wild varieties [8].

As a result of growing domestic and international demand, the cultivation area of honeyberry in northeastern China has expanded significantly. Because of the high cold tolerance of honeyberry, Heilongjiang and Jilin Province have become the largest production region in China [9]. The total seedling base area in Huanan Counties of Heilongjiang and Yongji Counties of Jilin is approximately 50 hectares, with an annual yield of about 5000 kg per hectare, generating considerable income for local farmers. However, in early 2022, a widespread root rot outbreak occurred in the “Beilei” cultivar, leading to a sharp decline in plant vigor and fruit production. No large-scale soil-borne diseases had previously been reported in honeyberry cultivation.

Root rot is commonly observed in field crops such as barley and wheat, and is primarily caused by fungi of the genus *Fusarium*. Several medicinal plants are also frequent hosts of *Fusarium* infections [10]. *Fusarium* spp. infect plants through combined hyphal penetration and enzymatic degradation of the cell wall. After infection, they secrete large amounts of mycotoxins that directly damage plant tissues, inducing and exacerbating root rot symptoms [11]. This results in browning of seedling tissues and, in severe cases, plant death [12]. Numerous control strategies for root rot have been reported in other plants [13]. Chemical control remains the primary approach, often involving fungicides that directly inhibit *Fusarium* growth. However, this may lead to the accumulation of chemical residues and soil pollution [14]. Alternatively, introducing biocontrol agents into the soil can suppress *Fusarium* growth, effectively controlling root rot with minimal environmental impact. Another sustainable control strategy involves enhancing plant disease resistance through genetic engineering, which, like biocontrol, is considered an environmentally friendly approach [15]. Nevertheless, research on root rot in honeyberry remains limited, hindering the development of effective management strategies.

Based on the aforementioned context, this study aims to test two verifiable hypotheses: First, the causal agent of root rot in the ‘Beilei’ honeyberry cultivar is a soil-borne fungus that can be isolated, purified, and shown to reproduce characteristic symptoms upon inoculation of healthy plants. Second, pathogen infection triggers a specific reprogramming of the root transcriptome in honeyberry, marked by the significant upregulation of key pathway genes associated with innate immunity, cell wall reinforcement, and phenylpropanoid metabolism. Hence, this study first isolated and identified (using morphological and molecular techniques) pathogenic fungi responsible for root rot in honeyberry. Subsequently, RNA-Seq transcriptomics was performed on inoculated plants to investigate the molecular mechanisms by which honeyberry responds to root rot disease. Identifying stress tolerance-related genes will lay the groundwork for breeding new varieties and adapting cultivation to diverse environments. Our findings enhance the understanding of honeyberry root rot disease and provide important insights for developing targeted biocontrol strategies against this emerging threat.

## 2. Results

### 2.1. Field Disease Symptoms

**Figure 1:**
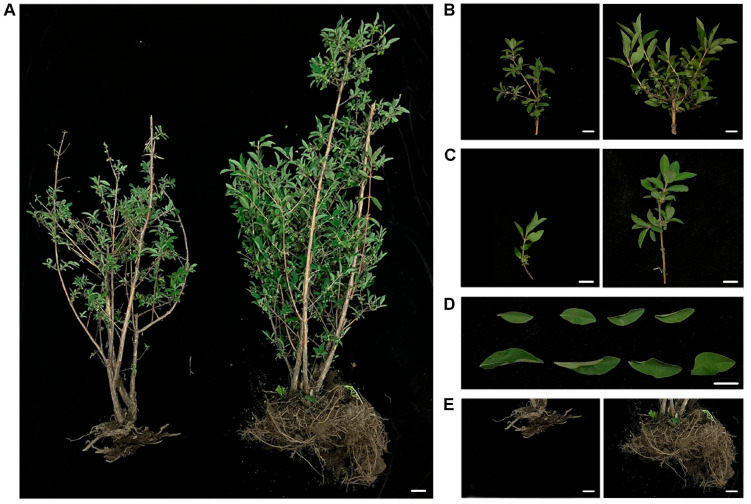
Phenotypic observation of honeyberry (*Lonicera caerulea* L.) infected with root rot disease. (**A**) Whole-plant phenotype; (**B**) main branch phenotype; (**C**) single branch phenotype; (**D**) leaf phenotype; (**E**) root phenotype. (**A**–**C**,**E**) Diseased plant on the left, healthy plant on the right. (**D**) Diseased plant on the top, healthy plant below. Scale bar: 2 cm.

In 2023, a large-scale disease outbreak occurred in the “Beilei” cultivar of *L. caerulea* in Yongji County, Jilin City, China. Over 90% of the affected plants showed a reduction in fruit yield to 30% of the normal level, severely impacting the local farmers. Diseased plants exhibit significantly weaker growth compared with healthy plants of the same age, including lower plant height (Figure 1A), noticeably fewer leaves and branches (Figure 1B,C), smaller leaves (Figure 1D), and a much less dense root system (Figure 1E). Overall, diseased plants displayed growth stagnation and decreased vigor; in severe cases, whole plants died.

### 2.2. Identification of Pathogenic Fungi

**Figure 2:**
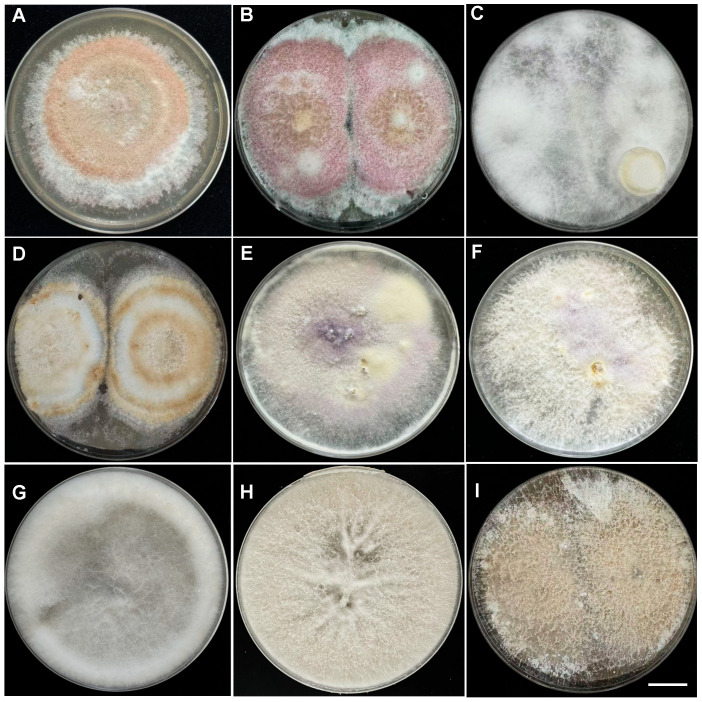
Phenotypic observation of nine fungal strains purified from honeyberry roots infected with root rot disease. The fungal species were assigned based on morphology and molecular observations (see also Table 2 and Table 3 and Figure 3). (**A**): *Fusarium* sp. (Colonies, 8 days, full coverage, plate), (**B**): *F. kyushuense* (Colonies, 7 days, full coverage, plate), (**C**): *F. solani* (Colonies, 7 days, full coverage, plate), (**D**): *F. equiseti* (Colonies, 10 days, full coverage, plate), (**E**): *F. annulatum* (Colonies, 7 days, full coverage, plate), (**F**): *F. foetens* (Colonies, 7 days, full coverage, plate), (**G**): *Trichoderma hamatum* (Colonies, 3 days, full coverage, plate), (**H**): *T. evansii* (Colonies, 4 days, full coverage, plate), and (**I**): *Ceratobasidium* sp. (Colonies, 4 days, full coverage, plate). Scale bar: 2.25 cm.

**Figure 3:**
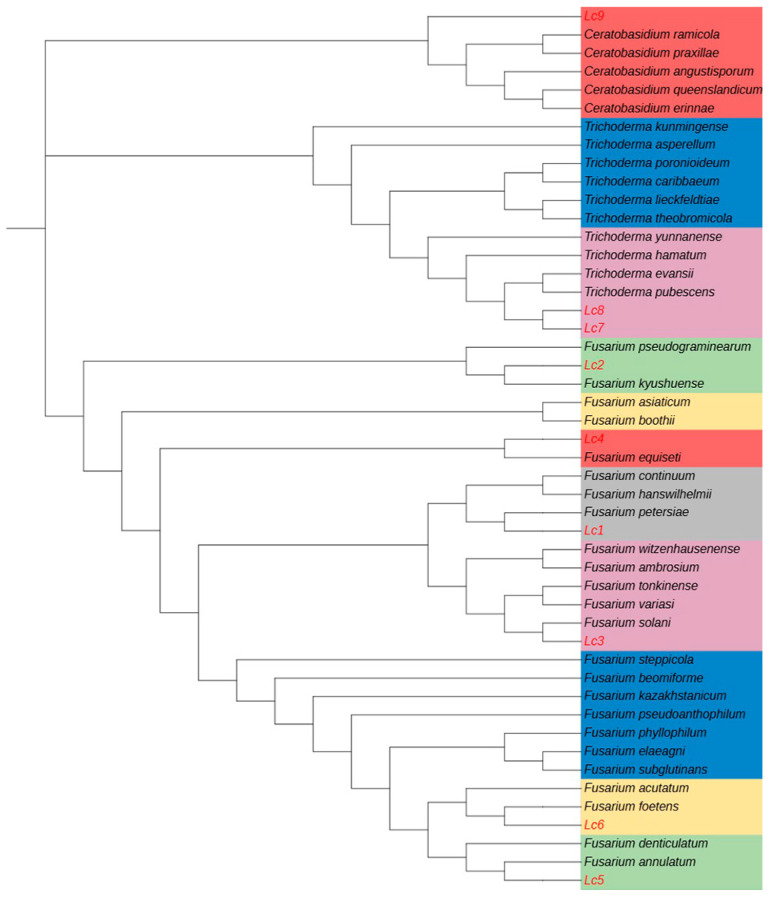
Phylogenetic analysis of nine fungal strains isolated from honeyberry roots infected with root rot disease based on the ITS gene sequence, using the Neighbor-Joining method. Novel isolates obtained in this study (Lc1–Lc9) are indicated in red. Bootstrap support values were calculated 1000 times. Reference sequences were uploaded from GenBank.

After isolation and purification of fungi from the roots of diseased plants, nine fungal strains with distinct morphologies were observed (named isolates Lc1–Lc9). These strains showed significant differences in colony morphology, colony color, hyphal state, and conidium size. Lc1–Lc6 showed raised hyphal margins and mostly irregular colony growth. Their conidia exhibited diverse morphologies, including falcate, elliptical, and ovoid shapes. Lc7 and Lc8 demonstrated white mycelia and nearly spherical conidia. Lc9 was characterized by light-yellow mycelia, and no conidia were observed (Figure 2 and Table 1). Based on ITS gene sequences of the strains and comparison with reference sequences, the nine fungi were classified into three genera: six strains belonged to the genus *Fusarium*, two to the genus *Trichoderma*, and one to the genus *Ceratobasidium*. *Fusarium* was the most frequently isolated genus, accounting for the highest proportion of all isolates. Phylogenetic analysis revealed that the Fusarium strains clustered into eight distinct clades, suggesting they may play a key role in the development of root rot (Figure 3). The nucleotide sequences of the ITS sequences from the fungal isolates in this study have been deposited in GenBank (Table 2).

### 2.3. Genus Fusarium as the Main Pathogenic Fungi

**Figure 4:**
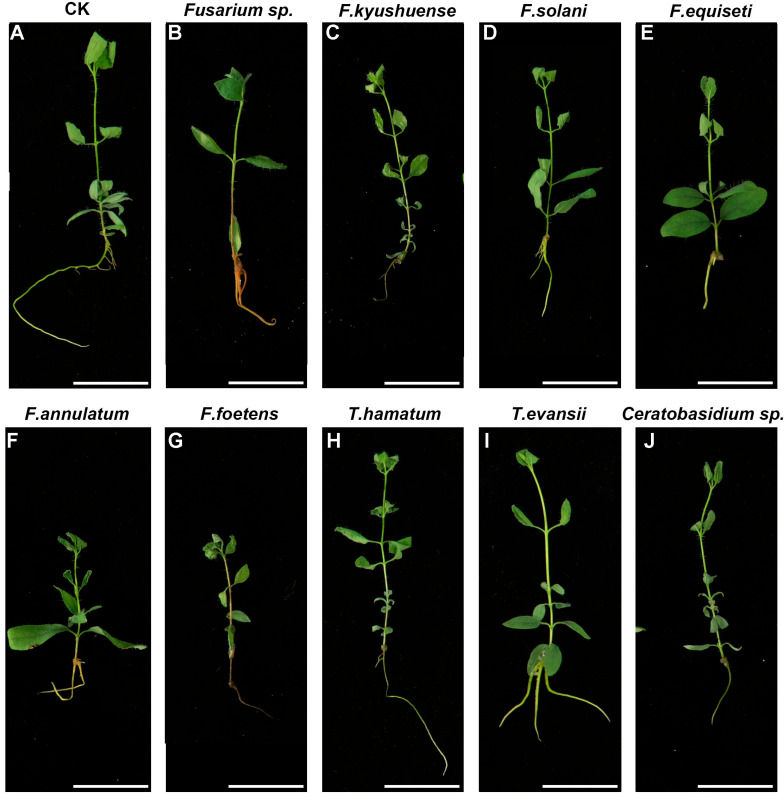
Phenotypes of honeyberry “Beilei” cultivar seedlings treated with fungal strains. (**A**): Honeyberry seedlings treated with sterile water (CK). (**B**–**J**): Honeyberry seedlings treated with mycelial suspensions of *Fusarium* sp., *F. kyushuense*, *F. solani*, *F. equiseti*, *F. annulatum*, *F. foetens*, *T. hamatum*, *T. evansii*, and *Ceratobasidium* sp., respectively. All seedlings were treated at 25 °C with a 16 h light/8 h dark photoperiod for 10 d. Scale bar = 2.25 cm.

A total of 10 d after inoculation, the root tips of seedlings treated with six *Fusarium* strains (*Fusarium* sp., *F. kyushuense*, *F. equiseti*, *F. solani*, *F. annulatum*, *F. foetens*) and *Ceratobasidium* sp. significantly turned black. Among these, *F. foetens* treatment caused the most severe inhibition of root growth. Thus, *Fusarium* was inferred to be the principal pathogenic genus. Notably, seedlings treated with the *T. hamatum* or *T. evansii* strains showed no difference compared with controls (Figure 4).

### 2.4. Phenotype and Electrolyte Leakage Analysis

**Figure 5:**
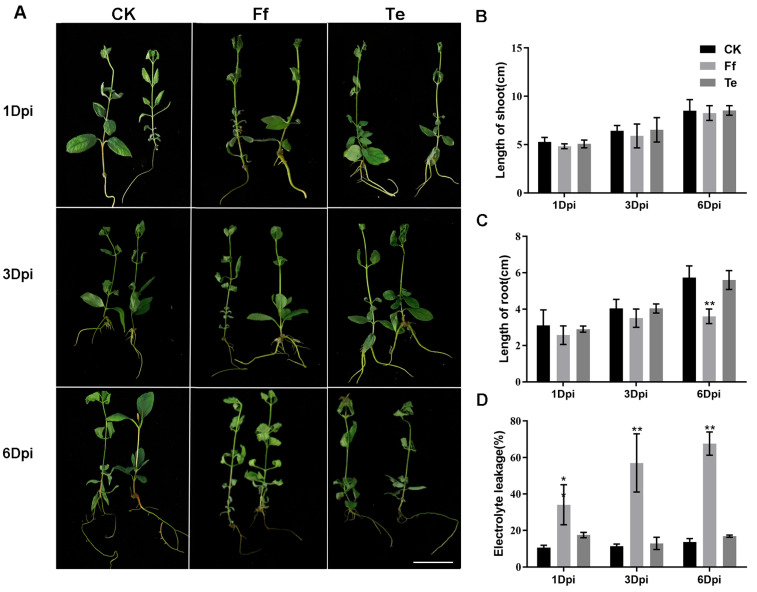
Phenotype and electrolyte leakage of honeyberry seedlings treated with *F. foetens* or *T. evansii*. (**A**) Phenotype images; (**B**) height of shoot; (**C**) length of root; (**D**) electrolyte leakage. Data are the mean ± SE of the mean from three replicates. * 0.01 < *p* < 0.05, ** *p* < 0.01. Scale bar = 2.5 cm.

Tissue-cultured seedlings of the honeyberry “Beilei” cultivar were treated with mycelial suspensions of *F. foetens* or *T. evansii*. At 6 dpi, the root length of the plants infected by *F. foetens* was significantly lower than that in the control (CK) group (Figure 5A–C, *p* < 0.05). The EL in the *F. foetens*-infected seedlings 1-, 3-, and 6-dpi was significantly higher than that in the CK group (Figure 5D, *p* < 0.05, *p* < 0.01). No significant difference in any measured parameter was observed between *T. evansii*-infected plants and controls.

**Figure 6:**
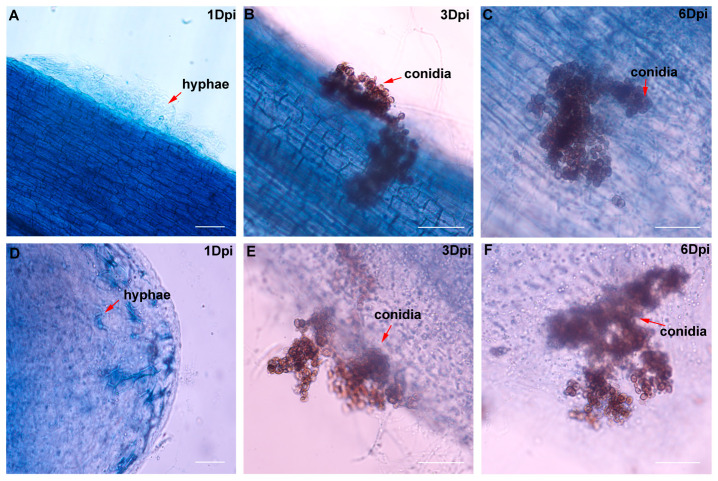
Morphological observation of *F. foetens* colonization of honeyberry roots. (**A**) Root meristem 1 dpi (d post-inoculation; 20× magnification); (**B**) root meristem 3 dpi (40×); (**C**) root meristem 6 dpi (40×); (**D**) root tip 1 dpi (20×); (**E**) root tip 3 dpi (40×); (**F**) root tip 6 dpi (40×). Scale bar = 10 μm.

To further observe the infection process of *F. foetens* in honeyberry, trypan blue staining was performed on roots at 1, 3, and 6 dpi. One day after *F. foetens* treatment, hyphae began to attach to the root meristem zone, and partial hyphal colonization was already observed at the root tips (Figure 6A,D). At 3 dpi, hyphae produced conidia and started to infect the root (Figure 6B,E). By 6 dpi, a large number of conidia had entered the plant roots (Figure 6C,F). Therefore, we chose to conduct transcriptome sequencing of *F. foetens*-infected plant roots at 1, 3, and 6 dpi.

### 2.5. Transcriptomic Analysis

**Figure 7:**
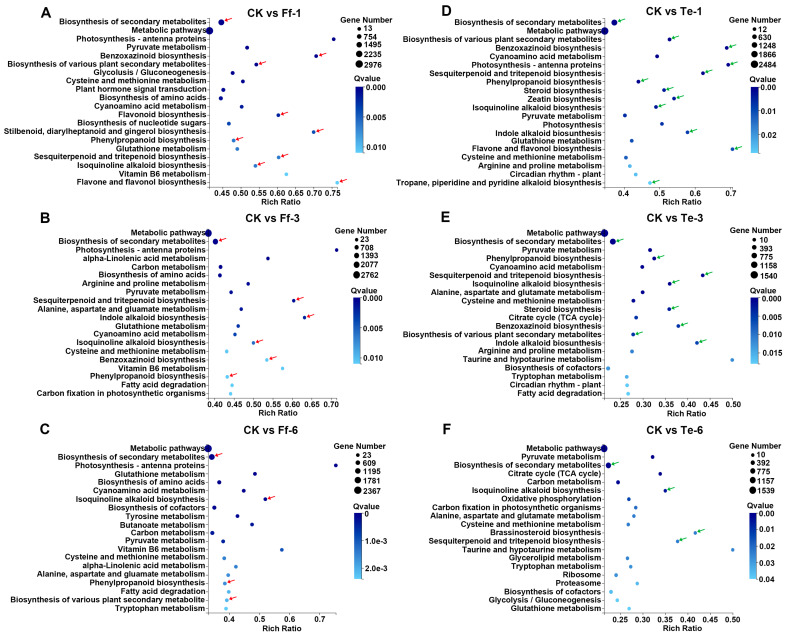
Kyoto Encyclopedia of Genes and Genomes (KEGG) pathway enrichment diagrams for honeyberry seedling roots exposed to infection by *F. foetens* or *T. evansii*. (**A**) CK vs. *F. foetens* infection, 1—dpi (Ff—1); (**B**) CK vs. *F. foetens* infection, 3—dpi (Ff—3); (**C**) CK vs. *F. foetens* infection, 6—dpi (Ff—6); (**D**) CK vs. *T. evansii* infection, 1—dpi (Te—1); (**E**) CK vs. *T. evansii* infection, 3—dpi (Te—3); (**F**) CK vs. *T. evansii* infection, 6—dpi (Te—6). This schematic was created based on the results of our transcriptome sequencing and pathway enrichment analysis, utilizing pathway information from the KEGG database.

**Figure 8:**
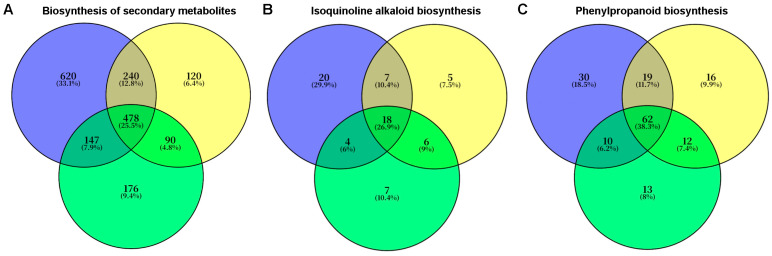
Venn diagrams showing numbers of differentially expressed genes (DEGs) from KEGG pathways in honeyberry seedling roots infected with *F. foetens*: 1–dpi (blue), 3–dpi (yellow), and 6–dpi (green).

Transcriptomic data were obtained for honeyberry seedlings without fungal infection (CK); for seedlings infected with *F. foetens* 1-dpi (Ff-1), 3-dpi (Ff-3), and 6-dpi (Ff-6); and for seedlings infected with *T. evansii* 1-dpi (Te-1), 3-dpi (Te-3), and 6-dpi (Te-6). KEGG pathway analysis revealed that in CK vs. Ff-1/3/6, DEGs were significantly enriched in “Biosynthesis of secondary metabolites”, “Phenylpropanoid biosynthesis”, and “Isoquinoline alkaloid biosynthesis” pathways (Figure 7A–C, marked with red arrows). In CK vs. Te-1/3/6, DEGs were significantly enriched in pathways such as “Biosynthesis of secondary metabolites”, “Isoquinoline alkaloid biosynthesis”, and “Sesquiterpenoid and triterpenoid biosynthesis” (Figure 7D–F, marked with green arrows). Venn diagrams identified distinct temporal gene expression patterns in response to infection of honeyberry root by *F. foetens*. In *F. foetens*-treated seedlings, 478, 18, and 62 DEGs were identified in analysis of CK vs. Ff-1/3/6 in “Biosynthesis of secondary metabolites”, “Isoquinoline alkaloid biosynthesis”, and “Phenylpropanoid biosynthesis” pathways, respectively (Figure 8). The raw sequencing reads generated by RNA-seq have been deposited in the NCBI Sequence Read Archive (SRA) under the accession number PRJNA1374457.

### 2.6. Differential Expression of Genes in Honeyberry Infected by F. foetens

**Figure 9:**
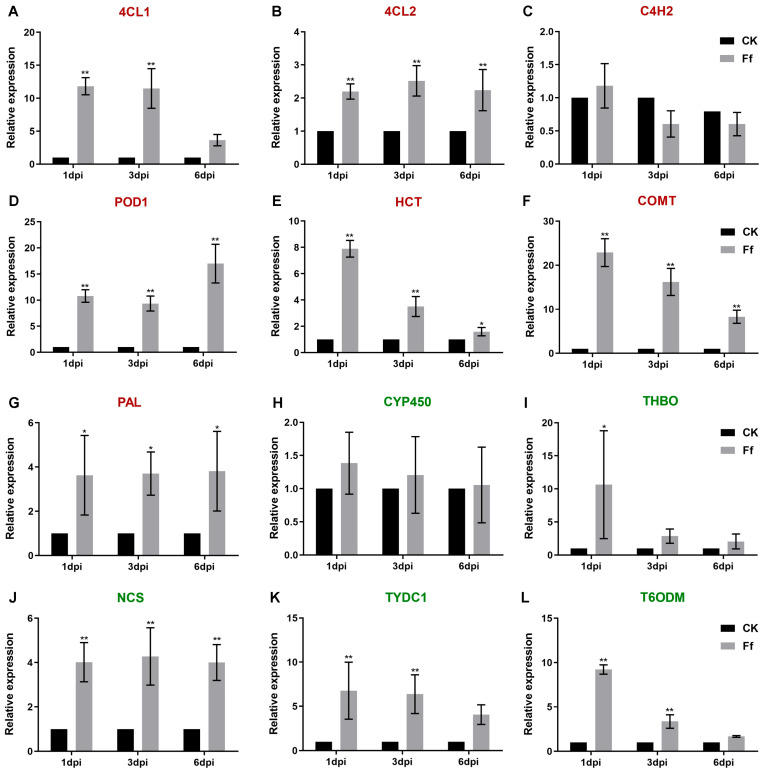
Relative expression levels of DEGs from the phenylpropanoid biosynthesis and isoquinoline alkaloid biosynthesis pathways in honeyberry seedlings infected by *F. foetens*. (**A**–**G**): DEGs (red font) from the phenylpropanoid biosynthesis pathway: (**A**) 4-coumarate-CoA ligase 1 (4CL1), (**B**) 4-coumarate-CoA ligase 2 (4CL2), (**C**) cinnamate 4-hydroxylase 2 (C4H2), (**D**) polyphenol oxidase 1 (POD1), (**E**) hydroxycinnamoyl transferase (HCT), (**F**) caffeic acid 3-O-methyltransferase (COMT), (**G**) phenylalanine ammonia lyase (PAL). (**H**–**L**): DEGs (green font) from the isoquinoline alkaloid biosynthesis pathway: (**H**) cytochrome P450 (CYP450), (**I**) tetrahydroprotoberberine oxidase (THBO), (**J**) S-norcoclaurine synthase (NCS), (**K**) tyrosine decarboxylase 1 (TYDC1), and (**L**) thebaine 6-O-demethylase (T6ODM). ** *p* <0.01, * *p* <0.05 (Student’s *t*-test).

The isoquinoline alkaloid biosynthesis pathway and the phenylpropanoid biosynthesis pathway are both secondary metabolite biosynthesis pathways. On infection of honeyberry seedlings by *F. foetens*, the expression levels of genes *4CL1*, *4CL2*, *POD1*, *HCT*, *COMT*, and *PAL* from the phenylpropanoid biosynthesis pathway were significantly higher than those in CK. Among them, the *COMT* gene exhibited the most pronounced induction, with expression level up to 23-fold that in controls at1-dpi (Figure 9A,B,D–G, *p* < 0.05). The activation of this pathway promotes the biosynthesis of phenolic acids (such as ferulic acid and caffeic acid), which exhibit direct antifungal activity, while also providing precursors for lignin synthesis. This enhances the cell wall through lignification, forming a physical defensive barrier [16]. However, the expression level of *C4H2* was similar in *F. foetens*-infected seedlings and CK (Figure 9C).

In *F. foetens*-infected seedlings, the genes *THBO*, *NCS*, *TYDC1*, and *T6ODM* of the isoquinoline alkaloid biosynthesis pathway were expressed at significantly higher levels than in CK (Figure 9I–L, *p* < 0.05). The *THBO* gene showed the greatest induction, with expression level about 10-fold that in the CK group at 1 dpi, although the expression level of this gene decreased with increasing time post-infection (Figure 9I, *p* < 0.05). The expression level of *NCS* was consistently about 4-fold that in CK (Figure 9J, *p* < 0.01). *TYDC1* and *T60DM* were expressed at approximately 7- and 9-fold the levels in the CK at 1 dpi, and their expression levels decreased at 3 and 6 dpi (Figure 9K, L, *p* < 0.01). The expression level of *CYP450* was slightly higher than that in the CK group at 1 and 3 dpi, but there was no difference compared with the CK at 6 dpi (Figure 9H).The upregulation of these genes indicates that honeyberry likely synthesizes isoquinoline alkaloids, such as berberine, which are often reported to function as both antifungal compounds and defense signaling molecules, contributing to the establishment of systemic chemical defense [17].

## 3. Discussion

Root rot is a worldwide family of plant diseases whose occurrence is closely linked to environmental conditions, soil physicochemical properties, and rhizosphere microbial community composition [18]. Major root rot pathogens include fungi from the genera *Fusarium* and *Phytophthora* [19]. *Fusarium* represents a large group of pathogenic fungi and ranks among the most destructive plant pathogens. *Fusarium* species have been reported to cause root rot in *American ginseng* [20], *Cinnamomum camphora* [21], and *soybean* [22]. In the present study, we identified *F. foetens* as a key pathogen of root rot in the honeyberry cultivar “Beilei” for the first time. This fungus rapidly infected the roots, causing root blackening and inhibiting aboveground growth. This finding aligns with recent reports of *F. foetens* causing severe root rot in a variety of plants, such as *sweet potato* [23], *tobacco* [24], *Lavandula angustifolia* [25], and *Schefflera arboricola* [26], providing further evidence for the broad host range of *F. foetens* and its ability to induce root rot in multiple plant species.

**Figure 10:**
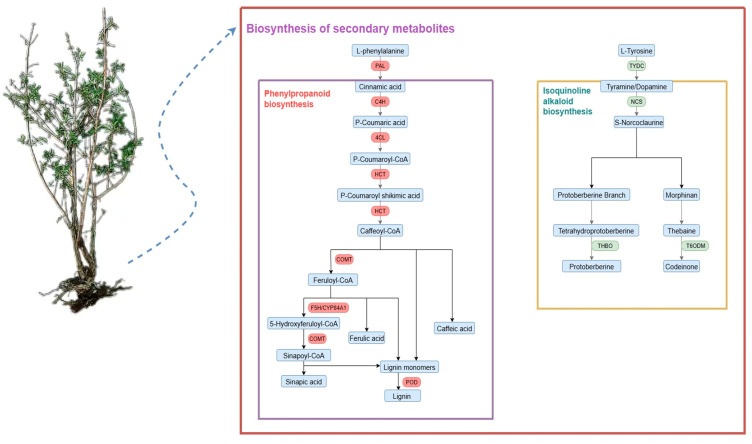
Schematic model of how honeyberry defends against root rot disease via biosynthesis of secondary metabolites.

In the present study, transcriptomic data revealed that treatment of honeyberry roots with *F. foetens* led to significant enrichment of genes in KEGG pathways such as “biosynthesis of plant secondary metabolites”, “phenylpropanoid biosynthesis”, and “isoquinoline alkaloid biosynthesis”, indicating that these pathways and their metabolites are a central mechanism by which honeyberry resists damage and disease caused by *Fusarium*. This finding is consistent with the general strategies plants employ to defend against *Fusarium* infection, as the production of secondary metabolites plays a crucial role in plant disease resistance. For example, disease-resistant cucumber cultivars enhance defense by accumulating secondary metabolites such as phenolic acids [27], and levels of ferulic acid and caffeic acid increase in banana following infection with *F. oxysporum* [28]. In this study, sustained high expression of the *PAL* gene in the phenylpropanoid biosynthesis pathway, together with upregulation of downstream genes including *4CL*, *HCT*, and *COMT*, collectively drove the synthesis of direct antimicrobial compounds such as ferulic and caffeic acids, as well as lignin precursors for cell-wall reinforcement. This finding is consistent with the results of a transcriptome study on healthy and diseased *Coptis chinensis* by Song et al., found substantial enrichment of genes involved in phenylpropanoid biosynthesis, plant hormone signal transduction, plant–pathogen interaction, and alkaloid biosynthesis pathways in the diseased plants. Key genes in phenylpropanoid biosynthesis, including *COMT*, *4CL*, and *PAL*, were identified as associated with the progression of root rot [29]. In the isoquinoline alkaloid biosynthesis pathway, upregulation of *TYDC1* likely enhanced the production of defensive metabolites such as berberine [30]. In addition, significant upregulation of genes including *NCS*, *THBO*, *T60DM*, and *CYP450* in this pathway would facilitate the formation of protoberberine alkaloids [31]. These alkaloids likely function as defensive signaling molecules, contributing to the activation of systemic plant defense mechanisms. The coordinated activation of these two pathways constitutes a multilayered disease resistance strategy in honeyberry, integrating chemical defense with physical barriers (Figure 10).

Based on the above mechanisms, this study points the way toward integrated management of honeyberry root rot in the future. In terms of genetic improvement, key defense genes such as *PAL*, *4CL*, *COMT*, and *TYDC1* can serve as important targets for molecular breeding, enabling the development of highly resistant cultivars through marker-assisted selection or gene-editing technologies. Second, defensive metabolites such as ferulic acid, caffeic acid, or berberine, after quantitative validation, are expected to be developed into metabolite biomarkers for rapid screening of resistant germplasm. In the field of biological control, *Trichoderma* species have shown potential as biocontrol agents against various *Fusarium* pathogens [32], including *F. solani* [33], *F. oxysporum* [34], and *F. verticillioides* [35]. For example, *T. atroviride* can produce cell wall-degrading enzymes that degrade the cell wall of *F. oxysporum* [36], and *T. asperellum* can induce hyphal deformation in *F. oxysporum*. The *Trichoderma* strain isolated and purified in this study may also serve as a potential biocontrol agent, though its efficacy against *Fusarium* requires further verification. Furthermore, as disease occurrence is also regulated by the soil environment, future management strategies could focus on optimizing the rhizosphere microenvironment to suppress pathogen growth.

## 4. Materials and Methods

### 4.1. Collection and Preservation of Diseased Samples

Honeyberry “Beilei” cultivar plants showing typical symptoms of root rot were randomly selected from the core seedling base of honeyberry in Yongji County, Jilin Province, China, in May 2023. For comparative analysis, one age-matched healthy plant and one diseased plant, both grown under identical natural conditions, were selected. Whole plants with intact roots were uprooted and placed in sterile fresh-keeping bags. Comparative analyses of plant height, leaf morphology, and root system architecture were performed between healthy and infected plants. Root tissues were aseptically excised and stored at 4 °C for subsequent pathogen isolation.

### 4.2. Isolation and Purification of Pathogenic Fungi

Root segments were rinsed under running water for 30 min to remove adhering soil, then blotted dry with sterile filter paper. Tissue blocks (5 × 5 mm) were excised from the junction of diseased root tissues. These tissue blocks were sequentially disinfected with 75% ethanol for 30 s and 2% available sodium hypochlorite (NaClO) solution for 3 min, and rinsed three times with sterile water. Finally, the disinfected tissue blocks were placed on potato–dextrose–agar (PDA) plates, and incubated in the dark at 25 °C for 3–5 d. Once colonies formed, the tips of hyphae were picked under a laminar flow hood for single-spore purification. This purification process was repeated three times until pure cultures with consistent morphological characteristics were obtained.

### 4.3. Morphological and Molecular Identification

After 7 d of culturing pure fungal colonies, macroscopic characteristics (form, elevation, texture, pigmentation, microconidia shape/width, and macroconidia shape/width) were observed and recorded under an optical microscope with an Olympus DP80 CCD camera. Genomic DNA of isolated fungi (0.05 g) was extracted using a Biospin Fungal Genomic DNA Extraction Kit (Hangzhou Bioer Technology Co., Ltd., Hangzhou, China), and internal transcribed spacer (ITS) gene sequences were amplified using primers ITS1 (5′–TCCGTAGGTGAACCTGCGG–3′) and ITS4 (5′–GCTGCGTTCTTCATCGATGC–3′). PCR products were sequenced by Guangzhou Ruqi Biotechnology Co., Ltd., Guangzhou, China. The obtained sequences were subjected to BLASTn searches against the NCBI database (http://www.ncbi.nlm.nih.gov/blast/Blast.cgi, accessed on 5 June 2024); the ITS sequences from fungal isolates were aligned with published fungal sequences. Multiple sequences were aligned using the program ClustalX 1.81. MEGA 5.0 software was used to construct a Neighbor-Joining phylogenetic tree.

### 4.4. Pathogenicity Assay

The nine isolated and purified potentially pathogenic fungal strains were activated on PDA plates. Mycelial plugs were taken using a sterile cork borer and inoculated into potato–dextrose broth, then cultured with shaking at 120 rpm at 25 °C for 5–7 d to prepare mycelial suspensions. The concentration of the suspensions was adjusted to 1 × 10^6^ spores/mL using a hemocytometer. Tissue-cultured honeyberry seedlings of the “Beilei” cultivar (obtained from tissue culture of wild varieties) with a uniform height of 5 cm were selected and immersed in 30 mL of mycelial suspension for 10 d. A control group (CK) was set up using 30 mL of sterile water. For each fungal isolate treatment, three seedlings were inoculated, and the experiment was repeated three times independently. All seedlings were cultured at 25 °C with a 16 h light/8 h dark photoperiod. Ten days post-inoculation, disease severity was assessed using a 0–4 scale based on the percentage of root tip area showing blackening: 0 (no symptoms), 1 (1–25%), 2 (26–50%), 3 (51–75%), and 4 (76–100%). Data presented are the mean values from three independent replicates.

### 4.5. Infection Kinetics and Histological Observation

Roots of the honeyberry “Beilei” cultivar were infected by *Fusarium foetens* suspension for 0, 1, 3, and 6 d post-inoculation (dpi). The root samples were bleached by heating in 10% KOH at 90 °C for 1 h, rinsed with distilled water, and acidified in 1% HCl for 4 min. Subsequently, the roots were stained with 0.05% lactophenol–trypan blue with lactic acid, glycerol, and deionized water (2:1:1, *v*/*v*/*v*) at room temperature for 24 h, then destained with lactoglycerol (lactic acid:glycerol:water 2:1:1, *v*/*v*/*v*) for 24 h. The processes of hyphal penetration, cortical colonization, and vascular bundle infection were recorded by using an upright fluorescence microscope (SOPTOP RX50, Hangzhou, China).

### 4.6. Phenotypic and Physiological Indicators

Tissue-cultured seedlings of the honeyberry “Beilei” cultivar were treated with 30 mL mycelial suspensions of *F. foetens* or *Trichoderma evansii* (as a control strain) (both at 1 × 10^6^ spores/mL). A control group was treated with 30 mL of sterile water. After 0, 1, 3, and 6 d, seedling plant height and root length were measured. In addition, plant tissues were placed in a 50 mL centrifuge tube, and 20 mL of deionized water was added to fully submerge the tissues. The tubes were vacuum-infiltrated for 15 min, followed by shaking at room temperature for 1 h. The conductivity was measured using a conductivity meter (Bante 510, Shanghai, China), with the sample recorded as C1 and the blank control as CK1. The tubes were then incubated in a boiling water bath for 15 min, cooled to room temperature, and the conductivity was measured again, with the sample recorded as C2 and the blank control as CK2. The relative electrolyte leakage (EL) was calculated using the formula: C (%) = (C1 − CK1)/(C2 − CK2) × 100.

### 4.7. RNA Extraction

Healthy tissue-cultured “Beilei” cultivar seedlings (with aboveground parts approximately 5 cm in height) were treated with 30 mL of mycelial suspension of *F. foetens* or *T. evansii*. Root tissues were collected after 1, 3, and 6 d. Samples were quickly frozen in liquid nitrogen and then sent to Beijing Genomics Institute, China, for transcriptome sequencing. Total RNA was extracted from the tissue using TRIzol^®^ Reagent (Thermo Fisher Scientific, Waltham, MA, USA). Then RNA quality was determined by 5300 Bioanalyser (Agilent Technologies, Santa Clara, CA, USA) and quantified using the NanoDrop 2000 spectrophotometer (Thermo Fisher Scientific, Wilmington, DE, USA). Only high-quality RNA sample [OD260/280 = 1.8~2.2, OD260/230 ≥ 2.0, RQN ≥ 6.5, 28S:18S ≥ 1.0, >1 μg] was used to construct the sequencing library.

### 4.8. Library Preparation and Sequencing

RNA purification, reverse transcription, library construction and sequencing were performed at Shanghai Majorbio Bio-pharm Biotechnology Co., Ltd. (Shanghai, China) according to the manufacturer’s instructions. The XX RNA-seq transcriptome librariy was prepared following Illumina^®^ Stranded mRNA Prep, Ligation (San Diego, CA, USA) using 1 μg of total RNA. Shortly, messenger RNA was isolated according to polyA selection method by oligo(dT) beads and then fragmented by fragmentation buffer firstly. Secondly double-stranded cDNA was synthesized with random hexamer primers. Then the synthesized cDNA was subjected to end-repair, phosphorylation and adapter addition according to library construction protocol. Libraries were size selected for cDNA target fragments of 300–400 bp use magnetic beads followed by PCR amplification for 10–15 PCR cycles. After quantification by Qubit 4.0, the sequencing library was performed on NovaSeq X Plus platform(PE150) using NovaSeq Reagent Kit(Illumina, San Diego, CA, USA).

### 4.9. Quality Control and De Novo Assembly

The raw paired end reads were trimmed and quality controlled by fastp [37] with default parameters. Then clean data from the samples were used for de novo assembly with Trinity [38]. To increase the assembly quality, all the assembled sequences were filtered by CD-HIT [39] and TransRate [40] and assessed with BUSCO (Benchmarking Universal Single-Copy Orthologs) [41]. The assembled transcripts were searched against the NCBI protein nonredundant (NR), Clusters of Orthologous Groups of proteins (COG), and Kyoto Encyclopedia of Genes and Genomes (KEGG) [42] databases using Diamond to identify the proteins that had the highest sequence similarity with the given transcripts to retrieve their function annotations and a typical cut-off E-values less than 1.0 × 10^−5^ was set. BLAST2GO [43] program(v6.0) was used to pobtainb GO annotations of unique assembled transcripts for describing biological processes, molecular functions and cellular components.

### 4.10. Differential Expression Analysis and Functional Enrichment

To identify DEGs (differential expression genes) between two different samples/groups, the expression level of each transcript was calculated according to the transcripts per million reads (TPM) method. RSEM (http://deweylab.github.io/RSEM/, accessed on 5 June 2024) [44] was used to quantify gene abundances. Essentially, differential expression analysis was performed using the DESeq2 (http://bioconductor.org/packages/stats/bioc/DESeq2/, accessed on 5 June 2024) [45]. DEGs with |log2FC| ≧ 1 and FDR < 0.05 (DESeq2) were considered significantly different expressed genes. In addition, functional-enrichment analysis including GO and KEGG were performed to identify which DEGs were significantly enriched in GO terms and metabolic pathways at Bonferroni-corrected *p*-value < 0.05 compared with the whole-transcriptome background. GO functional enrichment and KEGG pathway analysis were carried out by Goatools (https://github.com/tanghaibao/GOatools, accessed on 5 June 2024) and Python scipy software (https://scipy.org/install/, accessed on 5 June 2024).

### 4.11. Real-Time Quantitative PCR (RT-qPCR)

Total RNA extracted from root samples following infection with *F. foetens* was used as the template to synthesize 20 μL of cDNA using the Hifair^®^ III 1st Strand cDNA Synthesis Kit for qPCR (Yeasen, Shanghai, China). The levels of transcripts of key genes were measured by RT-qPCR using SupRealQ Ultra Hunter SYBR qPCR Master Mix (U+) (Vazyme Biotech Co., Ltd., Nanjing, China). The primers used are listed in Table 3. Gene expression was verified using a CFX96 Touch Real-Time PCR Detection System(Bio-Rad Laboratories, Hercules, CA, USA) for RT-qPCR. The 2^−ΔΔCt^ method was used to calculate the relative expression levels of target genes.

### 4.12. Statistical Analysis

Phenotype, physiological, and transcriptomic data are presented as the mean of three independent replicates. Bar graphs were constructed using GraphPad Prism (v5.03, GraphPad Software). The significance of differences between means was assessed using Student’s *t*-test and Microsoft Excel 2020. Images were combined using Adobe Photoshop CS.

## 5. Conclusions

We identified *Fusarium* as the causative agent of root rot in honeyberry and observed that secondary metabolite biosynthesis pathways are significantly activated upon *Fusarium* stimulation. Key genes within these pathways exhibit markedly upregulated expression levels, indicating an increase in the production of metabolites. These metabolites can alleviate the damage caused by *Fusarium* to the honeyberry root system. This study enhances our understanding of the pathogens involved in honeyberry root rot and provides a theoretical foundation for developing sustainable management strategies to combat this disease.

## Figures and Tables

**Table 1:** Tentative morphological descriptions of fungal strains purified from honeyberry roots infected with root rot disease.

Species	Colony Morphology				Microscopic Morphology	
	Form	Elevation	Texture	Pigmentation	Microconidia Shape/Width	Macroconidia Shape/Width
Lc1	Irregular	Raised	Velvety	Light brown to honey-colored, and the edges are rose-brown to brick red	Ovoid, ellipsoid to subcylindrical/4–8 × 2–3 µm	Curved fusiform/ 31 × 4 µm
Lc2	Irregular	Raised	Cottony	Red and white colors with a fluffy mycelium, and the edges are white	No Microconidia	Falcate to straight /38 × 5 µm
Lc3	Circular	Raised	Velvety	White mycelium	Ellipsoidal to ovoid /5–12 × 2–3.5 µm	Spindle-shaped to lanceolate/25 × 5 µm
Lc4	Irregular	Raised	Velvety	White mycelium which later produced a pale brown to dark brown pigmentation	Aseptate, hyaline, ovoid, fusiform (ellipsoidal), or slightly curved/8.2–13.8 × 2.0–4.5 µm	Slightly curved/18.1–41.6 × 2.5–4.9 µm
Lc5	Irregular	Raised	Velvety	White to purple	Obovoid with flatted base, reniform, fusiform/4.2–13.9× 0.9–3.8 μm	Ring shape and straight/12.5–65.6 × 0.9–4.9 μm
Lc6	Filamentous	Raised	Cottony	White to light purple	Ovoid to kidney shape/4.2–13.9 × 0.9–3.8 μm	Cylindrical/20.0–33.0 μm × 3.2–4.3 μm
Lc7	Circular	Flat	Velvety	White	Globalose, subglobose or broadly/3–4.5 × 2.5–3.5 μm	Ellipsoidal or obovoid/5–7 × 3–4 μm
Lc8	Circular	Flat	Cottony	White	Globalose or subglobose/3–4 × 2.5–3.5 μm	Ellipsoidal to Cylindrical/8–12 × 4–5.5 μm
Lc9	Irregular	Raised	Cottony	Light yellow	No Microconidia	No Microconidia

**Table 2:** Species with the closest phylogenetic relationships to the fungal strains isolated from honeyberry roots infected with root rot disease, identified based on ITS gene sequence alignment.

Closest Type Strain	Isolate Number	Similarity	Accession Number ^†^
*Fusarium* sp.	Lc1	91.18%	PX419195
*Fusarium kyushuense*	Lc2	96.96%	PX419178
*Fusarium solani*	Lc3	98.69%	PX421328
*Fusarium equiseti*	Lc4	98.25%	PX421525
*Fusarium annulatum*	Lc5	100.00%	PX421526
*Fusarium foetens*	Lc6	98.92%	PX421528
*Trichoderma hamatum*	Lc7	98.85%	PX421549
*Trichoderma evansii*	Lc8	99.55%	PX421563
*Ceratobasidium* sp.	Lc9	84.42%	PX421570

^†^ GenBank accession number of the ITS gene sequence obtained in this study.

**Table 3:** Sequences of primers used in this study.

Gene ID	Gene Symbol	Primers
Unigene34927-S21	4-coumarate--CoA ligase 1	F: GCGATTGCTAAAAGTCCGATT R: TCATTCCATAACCCTGTCCAAG
Unigene20850-S18	4-coumarate--CoA ligase 2	F: CAGAGAAGGAGAGTTGGCGG R: CACCACTTTCATCTCCTCACG
Unigene56302-S5	cinnamate 4-hydroxylase 2	F: TAGCCAAACAAGTCCTCCACAC R: ATGCTTTGACCATTACCCGTGA
Unigene31805-S17	polyphenol oxidase 1	F: CACCAAAATCACCAACACGCTC R: GTTGCCGCACAAGATAAAGAGC
Unigene12199-S5	hydroxycinnamoyltransferase	F: TTTCCATACGCCGAGTGTCTAC R: ATAAAAGGGAACTAAGGCTCGG
Unigene50287-S21	caffeic acid 3-O-methyltransferase	F: AGAAACACAAATCACCCCCCTA R: CACCATAGGTAAGACTGAGGCG
Unigene2458-S7	prephenate aminotransferase	F: TCTCTACAGACCTCACCACAACC R: CTGAAGCAAGAATCTGAATGAGG
Unigene10300-S19	cytochrome P450, family 706, subfamily A, polypeptide 2	F: TACCTCGCCTGCCCTACCTTA R: GTGTAGCCCATCACCTCGCAT
Unigene39881-S8	tetrahydroprotoberberine oxidase	F: TATCTGTGAGTGCGTTGGTGTC R: CAATGCCAAAGTTATGTCCTGC
Unigene57218-S10	S-norcoclaurine synthase	F: TAGAAGTGAAGGTGGGAGCAGG R: GCCAGTTGAAGGGTTCCATACA
Unigene46037-S12	Tyrosine decarboxylase 1	F: TCTAATAGCAGTGTTGCGGGA R: TTACTCCACCACCTTTCCCTG
Unigene32362-S5	thebaine 6-O-demethylase	F: GAGCAGTGACAAGCGAAACAAG R: TTGTAGGTTGCGGGTTTTTGTT

## Data Availability

The raw data supporting the conclusions of this article will be made available by the authors on request.
